# Thromboelastometry (ROTEM) Assessing Hypercoagulability in Patients Referred for Thrombophilia Screening

**DOI:** 10.1111/ijlh.14443

**Published:** 2025-02-20

**Authors:** Mazen Assar, Henning Nilius, Natalie Kearn, Wilma Hopman, Michael Nagler, Maha Othman

**Affiliations:** ^1^ Department of Biomedical and Molecular Sciences, School of Medicine Queen's University Kingston Ontario Canada; ^2^ Division of Internal Medicine, Department of Medicine University of British Columbia Vancouver British Columbia Canada; ^3^ Department of Clinical Chemistry Inselspital, Bern University Hospital, and University of Bern Bern Switzerland; ^4^ Kingston Health Sciences Centre Research Institute, and Department of Public Health Sciences Queen's University Kingston Ontario Canada; ^5^ School of Baccalaureate Nursing, St. Lawrence College Kingston Ontario Canada; ^6^ Clinical Pathology Department, Faculty of Medicine Mansoura University Mansoura Egypt

**Keywords:** blood coagulation tests, hypercoagulability, thromboelastometry, thrombophilia, venous thromboembolism

## Abstract

**Introduction:**

Thrombophilia, a blood coagulation disorder, poses risks of venous thromboembolism (VTE). Coagulation assays may not be sufficient to assess VTE risk and global assays such as Rotational Thromboelastometry (ROTEM) may add valuable information. We investigated ROTEM's capacity to detect hypercoagulability in patients undergoing thrombophilia screening, its potential impact on patient outcomes, and limitations.

**Methods:**

Comprehensive clinical, laboratory, genetic tests, and ROTEM (EXTEM and INTEM) were conducted for 356 patients referred for thrombophilia screening at an academic hospital outpatient unit. Hypercoagulability was identified as a shorter clot formation time (CFT), larger alpha angle (AA), and greater maximum clot firmness (MCF), and was compared in patients with and without VTE. Statistically this was analyzed using Mann–Whitney *U* and Chi‐square tests with *p* < 0.05 considered significant.

**Results:**

Among 356 patients, 64.6% had previous VTE, with 76.9% experiencing one event, 14.3% recurrent (35.6% unprovoked, 64.4% provoked). 22.5% of patients were on anticoagulation. Those with VTE history exhibited significant alterations in EXTEM and INTEM parameters compared to those without (*p* < 0.001), showing decreased CFT and increased AA and MCF. However, receiver operating characteristic curves for these variables indicated that none were able to discriminate between those individuals with and without thromboembolic complications.

**Conclusion:**

ROTEM does not appear to be a strong discriminatory test. However, it can detect hypercoagulopathy in patients referred for thrombophilia screening. Abnormal ROTEM may indicate a higher risk for recurrence. However, this can only be determined in prospective cohort studies.

## 1 Introduction

Venous thrombosis usually presents as deep vein thrombosis (DVT) and pulmonary embolism [1]. Thrombophilia refers to conditions associated with an increased predisposition to thrombosis. Hereditary thrombophilia denotes genetic disorders of specific hemostatic proteins, predominantly factor V Leiden mutation and the prothrombin gene mutation which constitute approximately 50%–60% of thrombophilia cases [1, 2]. The remainder is attributed to deficiencies in protein S, protein C, and antithrombin (formerly known as antithrombin III) [2].

Rotational Thromboelastometry (ROTEM) is a viscoelastic assay frequently used to either confirm hemostasis status or guide transfusions in individuals with hemostatic dysfunction, such as during major surgeries or traumatic events [3]. This test possesses the capacity to distinguish anomalies across all different clotting phases but lacks the capability to distinguish thrombocytopenia from platelet function abnormalities [3, 4]. The administration of viscoelastic testing, characterized by being a global hemostasis assay, is common either at the patient's bedside or within the operating theater [3, 5]. This assessment surveys the entire hemostatic continuum, from platelet activation and the coagulation cascade to clot formation and lysis [5]. Rather than recording a solitary endpoint like clotting time, ROTEM generates a comprehensive tracing, delineating the diverse stages of clotting over time, with distinct components of the tracing representing distinct aspects of the process, namely platelet function, clotting, and fibrinolysis [3]. It serves as a pivotal diagnostic tool for evaluating and monitoring coagulation status in surgical patients or those with bleeding disorders [3]. In contrast to conventional coagulation assessments like prothrombin time (PT) or activated partial thromboplastin time (aPTT), ROTEM offers a more holistic evaluation of coagulation by dynamically assessing the whole clotting process [3, 6]. It measures clotting time, clot strength, and fibrinolysis under varied stimuli, such as tissue factor, collagen, or thrombin, providing a more precise diagnosis of coagulation irregularities and guiding hemostatic interventions [6]. ROTEM has garnered widespread adoption in clinical practice [6]. Furthermore, ROTEM enables clinicians to tailor hemostatic therapies and gauge the effectiveness of interventions, such as transfusions and anticoagulant agents [7]. The other global viscoelastic test thromboelastography provides comparable function. However, correlation between both has been debatable [8].

Current evidence recommends against routine thrombophilia testing in unselected patients as several extensive prospective cohort studies conducted over the long term have indicated that the presence of an inheritable thrombophilia typically does not serve as a reliable predictor of recurrence [9]. An analysis of the MEGA study further revealed that undergoing testing for inherited thrombophilia did not lead to a reduction in the recurrence of venous thrombosis [10]. These findings led the authors to conclude that the increase in risk, taken in isolation, was modest and did not warrant an extended period of anticoagulation [10].

The purpose of this study is to examine the value of ROTEM in evaluating hypercoagulability during thrombophilia screening, its variations with VTE occurrence, and its limitations.

## 2 Methods

### 2.1 Study Design, Setting, and Population

This was a prospective cross‐sectional study. Consecutive patients referred for thrombophilia screening to a specialist outpatient unit of a tertiary hospital were included between January 2011 and September 2013. Inclusion criteria were: (1) referred for thrombophilia screening, (2) age above 18 years, (3) signed informed consent. No restrictions regarding previous thromboembolic events or the presence of underlying diseases applied. It is assumed that the Lucerne Cantonal Hospital received the majority of patients with thrombophilia tests during this time period. The hematology laboratory was the only laboratory in the region to offer special hemostasis tests. This hospital serves as a tertiary center for Central Switzerland covering both urban areas as well as rural communities. The study was approved by the local ethical committee and all patients signed informed consent (Kantonale Ethikkomission Luzern; #11001).

### 2.2 Collection of Data

Prior to the study's start, a detailed protocol was implemented to define all examination and data collection procedures. If the thrombophilia assessment was carried out after a venous thromboembolism, then at the time of completion of the initial anticoagulation. Detailed clinical characteristics were entered into a structured evaluation form, including the extent, triggers, and location of previous VTE, intermittent or persistent clinical risk factors, and family history. Clinical data were recorded by the responsible resident and reviewed by the attending physician. A trained study nurse and investigator checked the consistency of the data. A predefined set of routine laboratory parameters and ROTEM was performed on all patients.

### 2.3 Coagulation Tests

Venous blood was drawn using citrated tubes containing 1 mL trisodium citrate (0.106 mol L^−1^) for 9 mL of blood (Monovette, Sarstedt, Nümbrecht, Germany). A standardized protocol was used to ensure appropriate pre‐analytical conditions. Routine laboratory tests included PT, INR (Innovin; Siemens healthineers), PTT (Pathromtin SL, Siemens healthineers), thrombin time (Thrombin, Siemens healthineers), platelet count (XE 5000 (Sysmex AG, Horgen, Switzerland)) and special coagulation tests including coagulation factors II, V, VII X, fibrinogen levels (Thrombin, Siemens healthineers), D‐dimer (Vidas), ATIII (functional assay), protein C (functional), protein S (function; total/free) and APC resistance (russell viper venom) were conducted (STA‐R coagulometer, Stago, Glattbrugg, Switzerland). Thrombophilia genetic screen was conducted including FV Leiden and prothrombin gene mutation. Manufacturer's instructions were strictly followed.

### 2.4 ROTEM Analysis

Coagulability was assessed using the Delta ROTEM thromboelastometry analyzer within 15 min according to the manufacturer's instructions (Munich, Germany). The detailed methodology of ROTEM has been published previously [11, 12]. The test uses ~300 μL of citrated whole blood [12]. All samples were processed within less than 1 h after collection. Quality control was performed according to manufacturer's recommendation. Extrinsic rotational thromboelastometry (EXTEM), intrinsic rotational thromboelastometry (INTEM), and fibrinogen rotational thromboelastometry (FIBTEM) tests were assessed for each blood sample. The following parameters were evaluated. Clotting time (CT; s), Clot formation time (CFT; s), Alpha angle (AA; degrees), Amplitude at 10, 20, 30 min (A; mm), Maximum clot firmness (MCF; mm); Lysis Index at 30 min (LI30. %). Hypercoagulability was identified as a shorter CT, a larger AA and a greater MCF [13]. Manufacturer's reference range was used to assess ROTEM parameters. However, data were always compared between patients with and without previous VTE.

### 2.5 Statistical Analysis

Data were collected in an Excel file and imported into IBM SPSS (version 28.0 for Windows, Armonk, New York, 2022) for statistical analysis. The data were initially analyzed descriptively, including frequencies and percentages for categorical data, and means with standard deviations (SD) or medians with quartiles (IQR) for continuous variables. The underlying distributions of the continuous variables were assessed for normality using the Shapiro Wilk test. Associations between VTE and categorical variables were assessed using chi‐square tests or the Fisher's Exact test depending on cell sizes, while Student *t* tests and Mann–Whitney *U* tests were used to assess associations for continuous variables. A p‐value of < 0.05 was considered significant, and no adjustment was made for multiple comparisons. Receiver operating characteristic (ROC) curves were plotted for the major ROTEM parameters and the point on the ROC curve closest to sensitivity = specificity = 1 was considered the best cutoff value. The area under the curve (AUC) of the ROC curve was also generated, along with the associated 95% confidence intervals.

## 3 Results

Data was available for 356 patients (20 patients of the initial 376 had a diagnosis of cancer and were excluded from this analysis to avoid bias given cancer is a hypercoagulable state with high risk of VTE). Patients were subdivided into those who had VTE and those without a history of VTE. The median age of the whole tested population was 41 years (IQR 28, 55) years and 61.5% (219) were female. 64.6% (230) had at least one VTE, and 23% (82) of them had unprovoked VTE and are considered high‐risk patients for development of VTE. Additionally, 36% (124) had a positive family history and 22.5% (80) were started on anticoagulation. Ninety‐three patients (26.1%) had known thrombophilia traits with the most common being FV Leiden and prothrombin gene mutation. A history of arterial thromboembolism was present in 29 patients (8.1%). In regards to anti‐platelet therapy, 52 patients (14.8%) were receiving it, including 20 with a history of VTE. Details of clinical and demographic data based in the occurrence of VTE are provided in Table 1.

**TABLE 1:** Patients' demographics and clinical characteristics.

	VTE	No VTE	Total^a^	*p* value
Age (years)				
*n*	230	126	356	**< 0.001**
Median (IQR)	45 (33, 59)	33 (24, 44)		
BMI				
*n*	191	101	292	**< 0.001**
Median (IQR)	26 (23, 30)	23 (21, 26)		
Sex				
Male *n* (%)	107 (46.5)	30 (23.8)	137	**< 0.001**
Female *n* (%)	123 (53.5)	96 (76.2)	219	
Anticoagulation therapy				
Yes *n* (%)	72 (31.4)	8 (6.6)	80	**< 0.001**
No *n* (%)	157 (68.6)	115 (93.5)	272	
Antiplatelet therapy				
Yes *n* (%)	20 (8.7)	32 (26.0)	52	**< 0.001**
No *n* (%)	209 (91.3)	91 (74.0)	300	
Family history				
Yes *n* (%)	81 (35.8)	43 (36.4)	124	0.912
No *n* (%)	145 (64.2)	75 (63.6)	220	

*Note*: Patient demographics and clinical characteristics segmented by the presence of venous thromboembolism (VTE) among 356 individuals referred for thrombophilia screening. Variables include age, body mass index (BMI), sex, current anticoagulation therapy status, current anti‐platelet therapy status, and family history of VTE. Statistical significance (*p* value) is provided for each comparison. Bold indicates statistical significance (*p* < 0.05).

Abbreviations: BMI, body mass index; IQR, interquartile range; VTE, venous thromboembolism.

^a^ Totals may not equal 356 due to missing data.

Patients who had a history of VTE demonstrated significant increase in age and BMI, PT ratio, INR, TT, Fibrinogen, FV, and protein S (total and free) when compared to those who did not have VTE. There was no significant difference in any of the other coagulation factors, aPTT, platelet count, Protein C modified APC Ratio. Details of all coagulation tests and their reference ranges are provided in Table 2.

**TABLE 2:** Results of all coagulation tests in the 356 patients.^a^

	VTE	No VTE	Reference ranges [14, 15, 16]	*p* value
PT ratio (%)	230	126	0.8–1.2	**0.026**
Median (IQR)	91 (63,100)	92 (83,100)		
aPTT (s)				
*n*	230	126	21–30	0.940
Median (IQR)	33 (30, 37)	33 (31, 35)		
Fibrinogen (g/L)				
*n*	230	126	1.7–4.2	**< 0.001**
Median (IQR)	3.1 (2.6, 3.6)	2.8 (2.5, 3.2)		
Thrombin time (s)				
*n*	230	126	15.5–19.4	**< 0.001**
Median (IQR)	16.5 (15.8, 17.1)	16.0 (15.5, 16.7)		
D‐Dimer (ng/mL, FEU)				
*n*	211	105	< 190–500	0.051
Median (IQR)	267 (219, 486)	235 (219, 321)		
Factor II %				
*n*	226	121	78–130	0.152
Median (IQR)	101 (80, 114)	101 (92, 112)		
Factor V %				
*n*	226	121	65–140	**< 0.001**
Median (IQR)	108 (95, 125)	97 (87, 114)		
Factor VII %				
*n*	227	121	65–160	0.409
Median (IQR)	103 (67, 120)	101 (86, 120)		
Factor X %				
*n*	226	121	70–140	0.632
Median (IQR)	99 (74, 115)	97 (89, 110)		
AT %				
*n*	181	73	80–130	0.387
Median (IQR)	101 (94, 107)	102 (95, 110)		
Protein C chro %				
*n*	157	72	64–128	0.320
Median (IQR)	109 (93, 128)	107 (92, 118)		
Protein S free AG %				
*n*	193	99	27–61	**< 0.001**
Median (IQR)	82 (66, 97)	68 (59, 82)		
Protein S total %				
*n*	86	53	60–113	**0.014**
Median (IQR)	96 (81, 122)	87 (75, 102)		
Modified APC ratio				
*n*	178	85	> 2	**0.453**
Median (IQR)	2.4 (2.2, 2.6)	2.4 (2.3, 2.5)		
Prothrombin 20210				
*n*	153	65	N/A	N/A
(%)	(43.0)	(18.3)		

*Note*: Summary of coagulation test results for 356 patients categorized by VTE history. Tests include activated partial thromboplastin time (aPTT), fibrinogen levels, thrombin time, D‐Dimer, and several coagulation factors. Bold indicates statistical significance (*p* < 0.05).

Abbreviations: AG, antigen; aPTT, activated partial thromboplastin time; AT, antithrombin; VTE, venous thromboembolism.

^a^ Totals may not equal 356 due to missing data.

**FIGURE 1:**
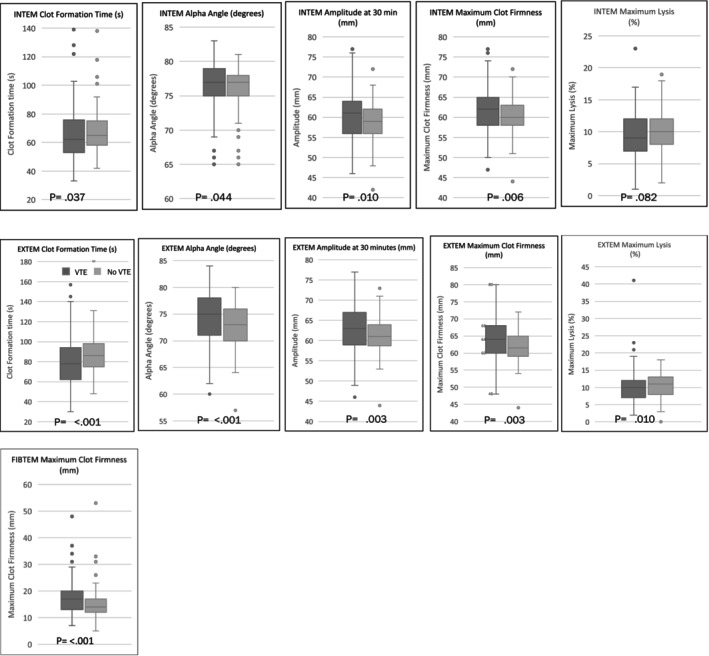
ROTEM parameters in the studied population. Graphical representation of ROTEM parameters INTEM and EXTEM including clot formation time (CFT), alpha angle (AA), and maximum clot firmness (MCF) among patients with and without a history of VTE. Results highlight significant differences in coagulation profiles between groups.

Patients who had a history of VTE demonstrated significant hypercoagulability as shown by various ROTEM parameters of EXTEM, INTEM and FIBTEM compared to those without VTE. Specifically, a statistically significant decrease in the CFT, an increase in AA, amplitude, MCF were observed. Details of the ROTEM measurements are provided in Figure 1.

**FIGURE 2:**
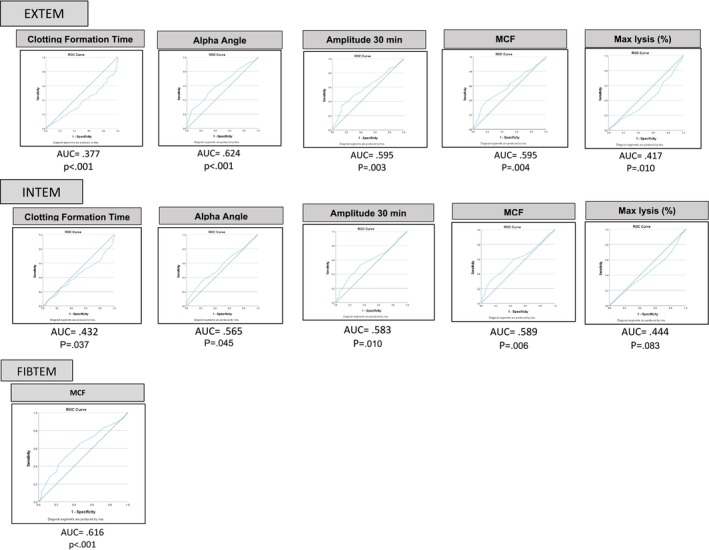
ROTEM: receiver operating curve. MCF, maximum clot firmness. Receiver operating characteristic (ROC) curves for ROTEM parameter. Clot formation time, alpha angle, and MCF assessed using EXTEM and INTEM assays in patients referred for thrombophilia screening. The area under the curve (AUC) values are displayed to evaluate the diagnostic ability of these parameters to discriminate VTE occurrence. The blue line represents patient data and the green line represents the line of zero predictive ability.

ROC curves were plotted for EXTEM and INTEM CFT, AA, Amplitude 30 and MCF. The AUC for these curves are shown in Figure 2. EXTEM angle had the largest area under the curve of 0.624. When defining the best cutoff as the point on the ROC curve closest to sensitivity = 1 and specificity = 1, none of the AUC for INTEM and EXTEM for any of the parameters were able to discriminate between those who had VTE and those who did not.

Table 3 depicts a comparative sub‐analysis of ROTEM parameters between those who had VTE and those without VTE within the two patient cohorts; those on anti‐coagulants and then those who were not on anti‐coagulants.

**TABLE 3:** Comparative analysis of ROTEM data between VTE and non VTE groups for the two patient cohorts; those on anticoagulation and those without anti‐coagulation therapy.

ROTEM parameters	Anticoagulation (*n* = 80)^a^			No anticoagulation (*n* = 272)^a^			Reference ranges
	VTE (*n* = 72)	No VTE (*n* = 8)	*p* value	VTE (*n* = 157)	No VTE (*n* = 115)	*p* value	
**INTEM clot formation time (s)**	58.0	67.5	0.536	64.0	65.0	0.239	105–285
Median (IQR)	(50.0, 72.0)	(49.3, 80.5)		(55.0, 77.0)	(58.0, 75.0)		
**INTEM alpha angle (degrees)**	78.0	76.5	0.569	77.0	77.0	0.220	50°–77°
Median (IQR)	(75.0, 80.0)	(74.0, 80.0)		(75.0, 79.0)	(75.0, 78.0)		
**INTEM amplitude at 30 min (mm)**	62.0	59.0	0.169	60.0	59.0	0.255	51–67
Median (IQR)	(59.0, 66.0)	(56.0, 63.8)		(56.0, 63.0)	(57.0, 62.0)		
**INTEM MCF (mm)**	63.0	60.0	0.144	61.0	60.0	0.185	54–73
Median (IQR)	(60.0, 66.0)	(57.5, 63.8)		(58.0, 65.0)	(58.0, 63.0)		
**INTEM ML %**	8.0	9.0	0.384	10.0	10.0	0.515	< 15%
Median (IQR)	(6.0, 11.0)	(8, 10.8)		(7.0, 12.0)	(8.0, 12.0)		
**EXTEM clot formation time (s)**	66.0	78.0	0.089	83.0	85.5	0.075	43–69
Median (IQR)	(52.0, 81.0)	(64.3, 97.0)		(70.0, 96.0)	(75.0, 98.8)		
**EXTEM alpha angle (degrees)**	77.0	75.0	0.163	74.0	73.0	**0.032**	50°–77°
Median (IQR)	(73.0, 79.0)	(70.8, 76.8)		(71.0, 77.0)	(70.0, 75.8)		
**EXTEM amplitude at 30 min (mm)**	65.0	62.0	0.404	62.0	61.0	0.070	53–70
Median (IQR)	(61.0, 68.0)	(58.8, 66.5)		(59.0, 66.0)	(59.0, 64.0)		
**EXTEM MCF (mm)**	66.0	63.0	0.279	63.0	61.0	0.091	55–72
Median (IQR)	(61.0, 69.0)	(59.5, 66.5)		(60.0, 67.0)	(59.0, 65.0)		
**EXTEM ML %**	9.0	9.5	0.672	10.0	11.0	0.096	< 15%
Median (IQR)	(7.0, 11.0)	(7.3, 10.8)		(7.0, 12.0)	(8.0, 13.0)		

*Note*: Bold indicates statistical significance (*p* < 0.05).

Abbreviations: MCF, maximum clot firmness; ML, maximum lysis.

^a^ Totals may not equal 356 due to missing data.

## 4 Discussion

The results of this study provide insight into the utility of ROTEM in assessing hypercoagulability in patients referred for thrombophilia screening. The findings demonstrate significant differences in ROTEM parameters between patients with a history of VTE and those without. Specifically, alterations in EXTEM and INTEM parameters were observed, indicating distinct coagulation patterns associated with VTE. However, the test does not appear to have discriminatory ability based on the ROC curve, indicating that further studies are still needed. Interestingly, no notable differences were observed in most ROTEM parameters when comparing participants who had VTE to those without VTE within the subset on anticoagulation therapy. Except EXTEM alpha angle which showed significant elevation in the VTE group versus no VTE within the subset not on anticoagulation, no other significant differences were seen (see Table 3), indicating that anticoagulation did not significantly influence the clotting dynamics in this cohort. Importantly, this manuscript does not specifically examine the role of viscoelastic testing in prospectively predicting thrombosis or tailoring anticoagulant therapy.

Previous studies showed ROTEM has been utilized in diverse clinical settings, including assessing coagulation, guiding transfusion therapy, monitoring anti‐coagulant therapy, and evaluating bleeding disorders. ROTEM detected hypercoagulability in obese patients and more precisely was significantly higher in I, II, and III degree obesity compared to the controls [17]. In COVID‐19 patients, ROTEM showed a hypercoagulable pattern which was more frequent in advanced disease groups and in patients with high IL6. This result suggests a potential role for ROTEM in determining thromboprophylaxis approaches for different severity groups, although further validation is required to confirm its effectiveness in guiding clinical decisions [18]. These studies show ROTEM can provide more specific information that can guide clinical assessments.

Regarding standard coagulation tests in our patient cohort, some tests showed significant differences, others showed insignificant differences. We believe the specificity of ROTEM in evaluating clot formation, strength, and fibrinolysis under various stimuli allows for a more comprehensive assessment of the coagulation process [19, 20]. This contrasts with traditional coagulation tests like PT and aPTT, which provide a more limited view of the clotting cascade [20, 21]. The inconsistency of significance in standard coagulation tests between the VTE and no VTE groups makes it difficult to draw conclusion on which tests can be recommended. While physicians frequently conduct investigations for inherited and acquired thrombophilia in patients following a VTE event, most current guidelines recommend against routine testing and to keep this only when the results is likely to impact the management decision [22, 23]. Moreover, results should be interpreted in the light of other clinical risk of VTE, such as recent surgery, hospitalization, malignancy, pregnancy, obesity and hormonal therapy.

Additionally, ROTEM parameters, particularly the alpha angle and maximum clot firmness, are primarily influenced by fibrinogen levels [24]. Elevated fibrinogen can lead to increased clot firmness and a steeper alpha angle, reflecting a hypercoagulable state [24]. Thus, while ROTEM can provide insights into clot formation and stability, it is important to consider that these results may be largely driven by fibrinogen concentration rather than being direct indicators of thrombotic risk. This highlights the need for caution when interpreting ROTEM results, as elevated fibrinogen could account for changes in these parameters without necessarily indicating a heightened risk of thrombosis.

The observed alterations in EXTEM and INTEM parameters between VTE and no VTE groups highlight the potential clinical relevance of ROTEM in assessing hypercoagulability in thrombophilia patients with a history of VTE, which may be useful to assess future risk of recurrence. The poor discriminating ability of ROTEM between VTE and Non VTE groups based on ROC curve and the fact that we have not used it to evaluate risk of recurrence remain to be an issue, thus, larger prospective cohort studies are needed to determine whether abnormal ROTEM translates into a higher risk for VTE recurrence and whether this could alter the clinical decision of anticoagulation intensity and duration.

## 5 Conclusion

In conclusion, this study contributes to the growing body of evidence supporting the clinical utility of ROTEM in hemostasis assessment. The data suggest that while ROTEM may detect hypercoagulability in patients with a history of VTE, its ability to predict thrombosis remains to be determined. ROTEM will unlikely replace the need for multiple standard coagulation tests to assess thrombosis. Perhaps a useful area for research would be to fully elucidate the role of ROTEM in assessment of VTE recurrence, based on hypercoagulability, as well as how to use the test for therapeutic decision‐making in this patient population.

## Author Contributions

H.N. and M.N. were responsible for collecting and providing all clinical and laboratory data. M.O. conceived and proposed the project, offering guidance in data interpretation. W.H. and N.K. conducted data analysis and interpretation. M.A. contributed to the project's conception, wrote the initial draft, and aided in data interpretation. N.K. wrote the initial draft. All authors reviewed, critiqued, and approved the manuscript.

## Ethics Statement

This study received ethics approval according to institutional policies, including patients' consent prior to starting the study.

## Conflicts of Interest

M.N. received research grants from Roche Diagnostics, Siemens Healthineers, Stago, and Bühlmann Laboratories, as well as lecture fees from Sysmex, Siemens Healthineers, Abbott, COR2ED, Werfen, Viatris, Silamed, Novartis, and Euroimmun, outside of the current work.

## Data Availability

Research data are not shared.
